# 2116 Exploring Müller cell-cone interactions in human fovea using 3-dimensional volume electron microscopy (EM)

**DOI:** 10.1017/cts.2018.72

**Published:** 2018-11-21

**Authors:** Ramya Singireddy, Kenneth R. Sloan, Jeff W. Lichtman, Christine A. Curcio, Dennis M. Dacey

**Affiliations:** 1 University of Alabama, Birmingham, AL, USA; 2 Harvard University, Cambridge, MA, USA; 3 University of Washington, Seattle, WA, USA

## Abstract

OBJECTIVES/SPECIFIC AIMS: Müller cells, radial glial cells of the retina, are the principal repository of xanthophyll pigment (lutein, zeaxanthin, meso-zeaxanthin), which are modifiable by diet and visible clinically by autofluorescence imaging. To understand the structural basis of xanthophyll visualization in vivo, we used 3-dimensional electron microscopic reconstruction of Müller cells surrounding one cone in a healthy human fovea. METHODS/STUDY POPULATION: From a 21-year-old male organ donor, dissected retinas were rejuvenated by oxygenated Ames medium then fixed in 4% glutaraldehyde. A tissue block 3.5 mm^2^ centered on the fovea was prepared for Automated Tape Ultramicrotomy (Kasthuri *et al.*, *Cell* 162: 648–661, 2015). From 1462 serial 65 nm horizontal sections, an area ~250×250 μm was imaged at 6 nm *xy* resolution. Images were stitched and aligned. TrackEM software on a pen display was used to trace, reconstruct, and display cone #5 (of 186) and its contacting Müller cells. RESULTS/ANTICIPATED RESULTS: Cone 5 is ensheathed by 2 types of Müller cells, outer and inner (Dacey, *ARVO*, 2016). The outer cell is first seen at the external limiting membrane (ELM) between cones 5 and 17. Moving inward from the ELM, it tightly wraps around cone 5’s fiber in a C-shape profile for 78 µm. This Müller cell also intermittently projects to neighboring cones, 2 of which were close to cone 5 at the ELM. As cone 5’s axon approaches the pedicle, it contorts into a corkscrew. The outer cell fluidly molds to this changing shape. At this level, this Müller cell doubles in volume to encompass not only cone 5, but also cone 17 and another Müller cell. In the final 17 µm of the block the Müller cell’s volume quickly dissipates as it sends a small projection towards the internal limiting membrane, eventually encasing an OFF midget bipolar cell also associated with cone 5. In contrast to this outer cell, an inner Müller cell adjoining cone 5 spans only 19 µm, interacting directly with cone 5 and the outer cell for 3.9 µm. DISCUSSION/SIGNIFICANCE OF IMPACT: Neural-glial relationships in a human fovea are visible through 3-dimensional volume EM. The volume of Müller cells in the fovea was impressive, consistent with a pivotal role in the health of cone photoreceptors and xanthophyll homeostasis. It is possible that individual glia also ensheath the post-receptoral neurons in a cone-driven circuit, supporting the concept that xanthophylls contribute to neural efficiency in vision.

